# Elevated thiamine level is associated with activating interaction between HIF-1α and SLC19A3 in experimental myopic guinea pigs

**DOI:** 10.3389/fmed.2025.1503527

**Published:** 2025-04-25

**Authors:** Zhongyu Ma, Jiawen Hao, Zhaohui Yang, Miao Zhang, Ruixue Zhang, Jizhao Xin, Bo Bao, Xuewei Yin, Hongsheng Bi, Dadong Guo

**Affiliations:** ^1^Shandong University of Traditional Chinese Medicine, Jinan, China; ^2^Affiliated Eye Hospital of Shandong University of Traditional Chinese Medicine, Jinan, China; ^3^Shandong Academy of Eye Disease Prevention and Therapy, Jinan, China; ^4^Medical College of Optometry and Ophthalmology, Shandong University of Traditional Chinese Medicine, Jinan, China; ^5^Shandong Provincial Key Laboratory of Integrated Traditional Chinese and Western Medicine for Prevention and Therapy of Ocular Diseases, Jinan, China

**Keywords:** experimental myopia, choroid, thiamine, SLC19A3, HIF-1α

## Abstract

**Background:**

The SLC19 gene family of solute carriers is a family of three transporter proteins with similar structures, of which SLC19A2 and SLC19A3 mediate thiamin transport; HIF is a transcriptionally active nuclear protein that is a key factor activated in hypoxic environments. Myopia is the most common eye disease that damages the visual health of adolescents, and currently, choroidal hypoxia is one of the prevailing doctrines of myopia, as well as the choroid as an ocular nutrient-supporting tissue, in which thiamine may play a role. This study aimed to investigate the process of thiamine changes in choroidal tissue of guinea pigs with negative lens-induced myopia (LIM).

**Methods:**

The right eyes of guinea pigs in the LIM group wore −6.0D lenses to model experimental myopia. Biological measurements of ocular parameters and choroidal thickness (ChT) were measured after 2, 4, and 6 weeks of modeling. Real-time fluorescence quantitative PCR and Western blot were used to detect the expression of SLC19A2, SLC19A3, and HIF-1α in the choroidal tissues of each guinea pig, ELISA was used to detect the changes of thiamine content in the choroidal tissues, and HE staining was used to observe the morphological changes of the choroidal tissues. Immunofluorescence and immunohistochemistry detected the expression of SLC19A3 and SLC19A3 in choroidal tissues at different modeling times, and protein immunoprecipitation and molecular docking verified the interactions between HIF-1α and SLC19A3.

**Results:**

Compared with the normal control (NC) group, the LIM group guinea pigs showed a significant increase in axial length and decrease in refractive error, as well as a thinning of choroidal thickness and loosening of tissue structure. In addition, the expression of SLC19A3 was higher than that of the NC group at 2 and 4 weeks, SLC19A2 was higher than that of the NC group at 4 weeks, and HIF-1α was higher than that of the NC group at 2, 4, and 6 weeks. Moreover, protein immunoprecipitation revealed a reciprocal relationship between HIF-1α and SLC19A3, and molecular docking showed their sites of action.

**Conclusion:**

The current study suggests that the choroidal tissue in myopic eyes is hypoxic and has metabolic abnormalities. Thiamine, a critical molecule for metabolism, may play a significant role in the process. Our findings indicate that changes in thiamine levels within the choroidal tissue are associated with elevated choroidal HIF-1α and activation of SLC19A3, which enhances thiamine transport. This suggests an adaptive regulatory mechanism for thiamine in myopia. Our research highlights thiamine as a potential target for pharmacological inhibitors and could lead to new insights into the study of the molecular mechanisms of myopia, as well as novel strategies for treating the disease.

## 1 Introduction

Myopia is the most common eye disease worldwide (1). From global data, approximately 1.95 billion people (28.3% of the world’s population) suffer from myopia and 277 million people (4% of the world’s population) suffer from high myopia. It is projected that by 2050, half of the world’s population (approximately 5 billion people) will suffer from myopia (2). Myopia, especially high myopia (<−6.00D), is associated with macular degeneration and retinal detachment (3), which is a serious threat to people’s lives and health, and has attracted a great deal of attention from society.

Thiamine, also known as vitamin B1, is a key cofactor involved in tissue energy and exists in the human body as free thiamine, thiamine monophosphate (TMP), thiamine phosphate (TPP), and thiamine triphosphate (TTP) (4). Thiamin plays an integral role in oxidative energy metabolism, ATP production and reduction, and cellular oxidative stress; therefore, changes in vitamin B1 at the cellular and tissue levels greatly impact cellular tissue physiology (5). Thiamine is an essential cofactor in carbohydrate and amino acid metabolism. Thiamin diphosphate (ThDP) is an active metabolite and cofactor for pyruvate dehydrogenase complex, *α*-ketoglutarate dehydrogenase complex, branched-chain α-ketoacid dehydrogenase complex, and the pentose phosphate pathway (cytoplasmic transketolase). In thiamin-deficient states, these enzymes limit the supply and cycling of the Krebs cycle, leading to reduced ATP synthesis, oxidative damage, and cell death (6). Thiamin deficiency leads to abnormalities in myocardial energy metabolism (7) and neurotransmission, most notably in the glutamatergic and GABAergic systems, resulting in a state of toxic neuroexcitation (8, 9). The mechanism of thiamine associated with metabolic abnormalities in guinea pigs has not yet been studied. In a rat model, thiamine supplementation was able to increase transketolase (TK), glutathione reductase, and Na/K adenosine triphosphatase activities, effectively reducing weight gain and plasma lipid levels in a high-fat diet model (10), as well as inhibiting endoplasmic reticulum stress and decreasing apoptosis to counteract glutamatergic toxicity (11). In addition, thiamine disulfide ameliorates insulin resistance in the liver by inhibiting gluconeogenic pathways (12). This suggests that there is some commonality between thiamine in humans and animals, both being able to influence disease progression by affecting metabolic processes and thus disease progression.

Humans and other mammals obtain vitamin B1 from exogenous sources, and in the diet, vitamin B1 exists in both free and phosphorylated forms; the latter form is converted to the free form by the action of small intestinal phosphatases before absorption, and then free thiamine is absorbed through a specific carrier-mediated mechanism that involves two transporters: thiamine transporter-1 (THTR-1) and thiamine transporter-2 (THTR-2), which are encoded and produced by SLC19A2 and SLC19A3, respectively (13). The choroid, which is the posterior part of the uvea between the retinal pigment epithelium and the sclera, is a highly vascularized layer that provides nutrients and oxygen to the retina and is uniquely situated to transmit retinal-derived signals to the sclera, which in turn affects ECM synthesis and changes in eye size (14). In this experiment, by establishing a negative lens-induced myopia guinea pig model, we explored the changes of thiamine and its transporter protein expression in myopic choroidal tissues, to provide a theoretical basis for the molecular mechanism of myopia.

## 2 Materials and methods

### 2.1 Experimental animals

These animals were subjected to strict animal ethics and welfare treatment. This study was approved by the Experimental Animal Ethics Review Committee of the Affiliated Hospital of Shandong University of Traditional Chinese Medicine (AWE-2022-055) and strictly adhered to the Animal Research Statement for Vision and Ophthalmology (ARVO) principles. Sixty healthy British tricolor short-haired guinea pigs (male, 2 weeks old, purchased from Jinan Jinfeng Laboratory Animal Co., Ltd., Shandong, China) were selected, weighing 100–120 g. Before the experiments, all the animals were examined to exclude ocular diseases, such as cataracts and corneal diseases. The experimental animals were placed in a room with a temperature of 25°C ± 2°C and a circadian rhythm of 12 h/12 h (alternating day and night). All guinea pigs were provided with an adequate nutritious diet and water daily.

The guinea pigs were randomly divided into a normal control (NC) group (*n* = 30) and a lens-induced myopia (LIM) group (*n* = 30). In this study, all animals in the NC group were left untreated, and guinea pigs in the LIM group were covered with a − 6.0 D lens over the right eye to induce experimental myopia. In contrast, the untreated left eye served as a self-control. In this study, all spectacles were examined twice a day, morning and evening, and the lenses were replaced immediately if they were blurred or detached.

### 2.2 Main instruments and reagents

Aspheric resin lenses (Jiangsu NuShang Optical Spectacles Co., Ltd.); YZ24 banded light microscope (Suzhou LiuLiu Vision Technology Co., Ltd.); ophthalmic A-type ultrasonic instrument (Quantel Medical, France); Light Cycler^®^ 480II real-time fluorescence quantitative PCR instrument (Roche, Switzerland); chemiluminescent solution (Vazyme, Nanjing, China); goat Anti-immune IgG secondary antibody (SparkJade, Shandong, China); goat anti-mouse IgG secondary antibody (SparkJade, Shandong, China); SLC19A2, SLC19A3 primary antibody (Bioss, Beijing, China); HIF-1α primary antibody (Santa Cruz biotechnology, USA); Protein A and G magnetic beads (Vazyme, Nanjing, China).

### 2.3 Biological data measurements

#### 2.3.1 Refractive error measurement

The guinea pigs in each group were subjected to refractive index examination after 2, 4, and 6 weeks of modeling. Before measurement, the pupils were dilated with cyclopentolate hydrochloride eye drops (Alcon, Geneva, Switzerland) in a dark room, 1 drop each time at 5 min intervals, and waited for 35 ~ 45 min for refraction after 3 drops. The working distance of the detector was set at 50 cm, and the average value was taken as the refraction of the guinea pigs after 3 repeated measurements using the ball-and-column method.

#### 2.3.2 Measurement of axial length

Ophthalmic A-type ultrasonography was used to measure the axial length of the eyes of the guinea pigs in each group after 2, 4, and 6 weeks of modeling. Before measurement, surface anesthesia was applied to the conjunctival sac by placing a drop of oxybuprocaine hydrochloride (Santen Pharmaceutical, Osaka, Japan) in the conjunctival sac. The parameters of Ophthalmology A ultrasonic instrument were set as follows: anterior chamber propagation speed of 1,557 m·s^−1^, lens propagation speed of 1,723 m·s^−1^, and vitreous propagation speed of 1,540 m·s^−1^. The ultrasonic probe was lightly attached to the corneal apex of the guinea pigs during the measurement, and the average value was taken as the final length of the axial length of the eyes after repeated measurements 10 times, which was performed by the same professional optometrist.

### 2.4 Choroidal thickness

Measurement of choroidal thickness (ChT) is described in our previous article (15). Briefly, the relevant parameters of ChT were determined by scanning the center of the guinea pig optic disc. Using spectral domain OCT (Heidelberg Engineering, Heidelberg, Germany). As shown, the upper border of the choroid is the epithelium of the outer surface of the retina, the lower border is the inner surface of the sclera, and the optic disc is the center of the reference. In the literature (16), two concentric circles of radius 600 μm and 1,050 μm were defined. The average ChT was obtained from eight locations in four quadrilateral areas by selecting the area to measure the ChT near the intersection of the two concentric circles with the blue line. In addition, we performed a correlation analysis of the ocular axial length with the choroidal thickness.

### 2.5 Real-time fluorescence quantitative PCR

After 2, 4, and 6 weeks of myopic induction, six guinea pigs were randomly selected from each group and anesthetized to death by intraperitoneal injection of 40 g-L^−1^ pentobarbital, the eyeballs were extracted, rinsed with sterile cold PBS, the eyeballs were cut open about 2 mm behind the corneal limbus, the outer wall of the eyeballs was scraped gently, and the choroidal tissues were separated and stored in the freezing tubes (NEST, Wuxi, China) at −80°C after quick freezing in liquid nitrogen. The total RNA of retinal tissue was extracted using a modified tissue/cellular RNA extraction kit (Shandong Sparkjade Biotech. Co., Ltd., Jinan, China). Subsequently, the mRNA expression levels of SLC19A2 and SLC19A3 were detected by real-time fluorescence quantitative PCR (Q-PCR) after reverse transcription into cDNA. The primer sequences of each gene were designed using DNAStar software (Table 1), and the relative mRNA expression of SLC19A2, SLC19A3 and HIF-1α in the retinal tissues of guinea pigs in each group was quantified using the 2^-ΔΔCt^ method.

**Table 1:** Primer sequences of the target genes.

Gene	Primer sequence
SLC19A2	F:5′- TCCTCCCGCCCCCTGCTCTGCT −3′ R:5′- GAAACGGCCTCCACCCCACCATTG −3′
SLC19A3	F:5′- GGTTATCGGCGCTGGCTCTC -3′ R:5′- TACTTCTTTCCGGCATTTGACTGA −3’
HIF-1α	F:5′- TTCTGCAAGCCCCCAAAGTGTGAG −3′ R:5′- CGCTGTATGGTGGTGATGTTGTGG −3′
GAPDH	F:5′- CTGACCTGCCGCCTGGAGAAACC -3′ R:5′- ATGCCAGCCCCAGCGTCAAAAGT −3′

### 2.6 Western blot detection of protein expression level

After 2, 4, and 6 weeks of modeling, 6 guinea pigs in each group were randomly selected to isolate the choroidal tissues, and the protein was extracted to determine the protein concentration. Furthermore, 10% SDS-PAGE electrophoresis (Shandong Sparkjade Biotech., Co. Ltd., Jinan, China) was performed in each group, and then the samples were transferred to the PVDF membrane, which was closed with 50 g-L^−1^ skimmed milk powder for 2 h, washed with TBST 3 times, and then anti-SLC19A2 antibody (1:1000), anti-SLC19A3 antibody (1:1,000), anti-*β*-actin antibody (1:4000), and mouse anti-HIF-1α antibody (1:500) were added. After 3 washes with TBST, the antibody was incubated for 1 h at 4°C on a shaking table (1:15,000) and then washed 4 times with TBST for chemiluminescence development. The gray value of the bands was calculated using CAPT software, and the protein loading on the membrane was corrected by the gray value of the internal reference *β*-actin band (see Table 2).

**Table 2:** Detailed information for primary antibodies.

Antibody	Dilution ratios	Antibody brands	Catalog number	Brand information
Beta actin	1:4,000	Service bio	GB15003-100	Wuhan, China
SLC19A2	1:1,000	Bioss	bs-10738R	Beijing, China
SLC19A3	1:1,000	Bioss	bs-8702R	Beijing, China
HIF-1α	1:500	Santa Cruz	sc-10790	CA, USA

### 2.7 Detection of thiamine content by ELISA

The procedure for guinea pig choroidal protein extraction was the same as that in section “2.5 Western blot for protein expression level.” The thiamine level in the guinea pig choroid was detected using the appropriate ELISA kit (Jianglai Biotechnology Co., Ltd., Shanghai, China) according to the manufacturer’s instructions.

### 2.8 Histopathological staining

At 6 weeks of induced myopia, three guinea pigs in each group were randomly selected for histopathological staining. The guinea pigs were euthanized by intraperitoneal injection of 4% pentobarbital, and then the nucleus of the eyeball was removed and the periocular tissues were debrided. The eyes were immediately fixed with 4% paraformaldehyde, then routinely dehydrated, paraffin-embedded, sectioned as 5-μm sections, and stained with hematoxylin and eosin (H&E).

### 2.9 Immunohistochemistry and immunofluorescence staining

Sections were placed in citrate antigen recovery buffer (pH 6.0) to take antigen, then 3% hydrogen peroxide solution was used to block endogenous peroxidase, followed by supplementation with 3% BSA for 30 min, and then dropwise primary antibody was incubated at 4°C overnight. In addition, the secondary antibody was incubated at room temperature for 50 min, followed by DBA for controlled color development time under a microscope, and then with 3,3 ‘stained-diaminobenzidine (3,3 DAP, Sigma-Aldrich, Germany) for 5 min at 37°C. Images were captured by fluorescence microscopy (Nikon, Eclipse, 55i, Tokyo, Japan).

Sections were placed in EDTA antigen recovery buffer for antigen recovery, rinsed in PBS (5 min × 3), and BSA was added dropwise; blocking was performed for 30 min, and then primary antibody was added and incubated at 4°C overnight. The secondary antibody was then incubated for 50 min at room temperature, and then the DAPI stain was incubated for 10 min at room temperature and quenched with an autofluorescence quencher. The autofluorescence quencher was quenched for 5 min. Sections were slightly spin-dried and then slightly spin-dried, sealed with anti-fluorescence quenching mounts, and visualized with a fluorescence microscope. The observation was performed with a fluorescence microscope (Nikon, Eclipse, 55i, Tokyo, Japan).

### 2.10 Protein immunoprecipitation and molecular docking

Fresh guinea pig choroidal cells were lysed using RIPA lysis solution (New Cell & Molecular Biotech, P70100). The lysate was then centrifuged at 12,000 rpm for 15 min at 4°C. The supernatant was collected into new tubes, and the protein concentration was determined by adding 10 μL of protein A and G magnetic beads (Vazyme, PB101-01) to each tube. The magnetic beads were washed with pre-cooled PBS, and the proteins were added and incubated for 60 min at 4°C with rotation to remove non-specific proteins.

Next, a monoclonal antibody (1–5 μg) specific to the target protein was added and incubated for 2 h at 4°C. The beads were then washed with pre-cooled PBS. As a control, a non-specific immunoglobulin G (IgG) antibody was also included; this was mixed gently and incubated overnight at 4°C.

After overnight incubation with the antibody, the beads were washed with pre-cooled PBS, and the protein samples were added. These were incubated at 4°C for 3–4 h. Following this incubation, the beads underwent 3–4 washes with pre-cooled immunoprecipitation (IP) lysis buffer. The precipitate was then resuspended by adding 1 × SDS sample buffer, vortexing, shaking, and heating the sample to 95–100°C for 5 min. Finally, the samples were loaded onto 10% SDS-PAGE gels (Shandong Sparkjade Biotech Co., Ltd., Jinan, China) for protein blot analysis.

The protein models used for docking were HIF1-*α* (Uniprot ID: H0V563), and SLC19A3 (Uniprot ID: H0VQG5). HDOCK SERVER^1^ was used to perform protein–protein molecular docking (17). Pre-processing of proteins (deletion of water molecules and redundant ligands, addition of hydrogen atoms) was accomplished using PyMol 2.4. Docking Score, Confidence Score, and Ligand RMSD were used as the evaluation criteria for docking, and the docking results were set to output the 10 best docking positions. The model with the highest score was selected as the best docking model. Finally, we visualized the docking results using Pymol 2.4 software. In this way, we were able to visualize the binding between the proteins.

### 2.11 Statistical analysis

Statistical analysis was conducted using SPSS Statistics 23.0 (IBM, Chicago, IL, United States). Within-group comparisons: a repeated measures ANOVA was applied to compare refractive parameters of the same eyes across multiple treatment time points, followed by Bonferroni-corrected *post hoc* tests if the overall effect was significant (*p* < 0.05). Between-group comparisons:, independent samples t-tests were used for comparisons between the right eyes of the experimental (LIM) group and the control (NC) group, as only two independent groups were involved. For gene expression differences between the NC and LIM groups, independent samples t-tests were also employed, given the normal distribution of data (verified by Shapiro–Wilk test, *p* > 0.05) and equality of variances (Levene’s test, *p* > 0.05). All reported *p*-values were adjusted for multiple comparisons using the Bonferroni method, with statistical significance set at *α* = 0.05.

## 3 Results

### 3.1 Myopia induces physiological changes in the eye

#### 3.1.1 Changes in refraction and axial length of eyes

**Figure 1:**
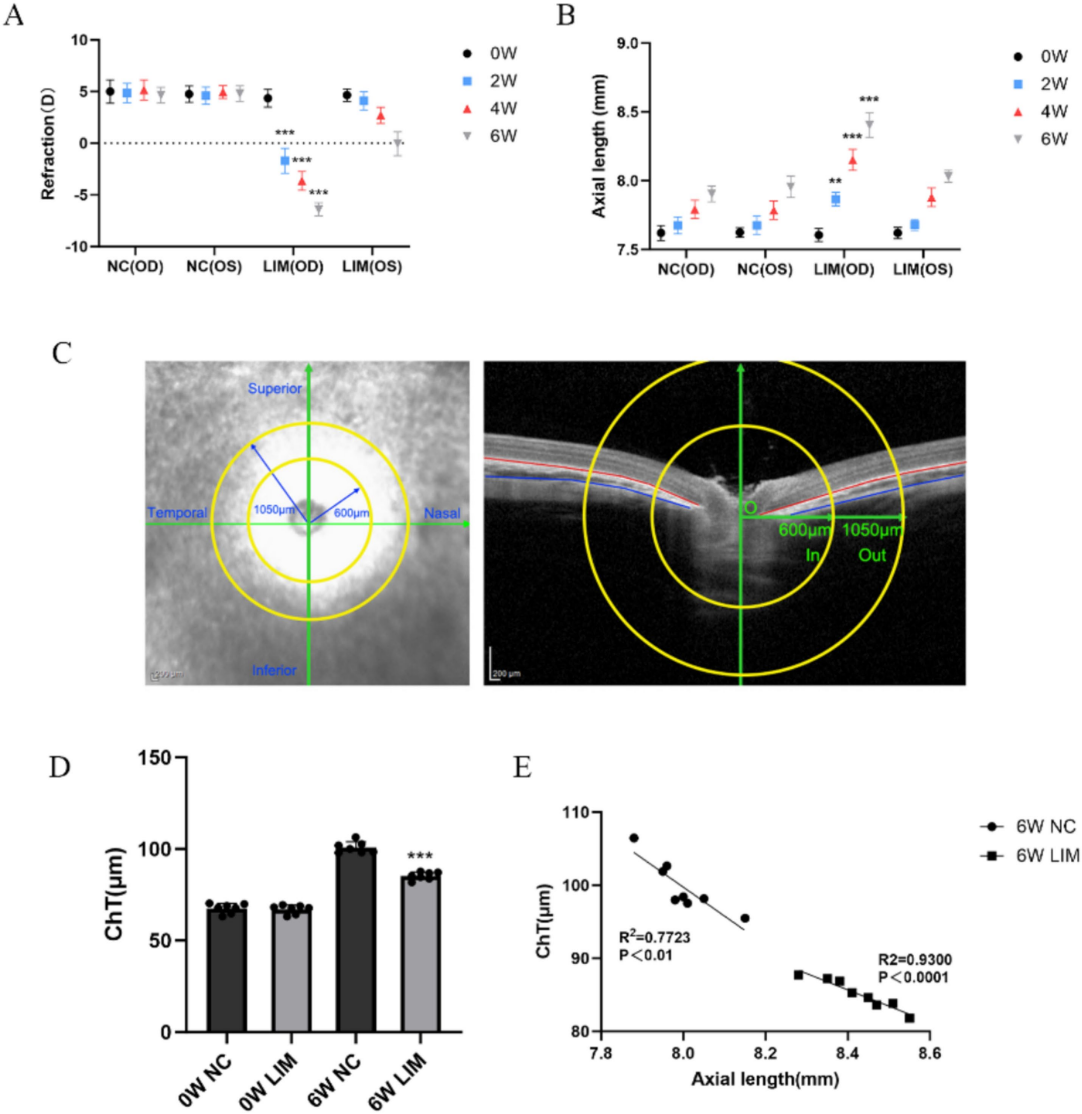
Changes in refraction and axial length of the eyes among the NC and LIM groups after 2, 4, and 6 weeks of myopic induction. **(A)** Refraction and **(B)** axial length. *N* = 8, ***p < 0.001 vs. the NC group. Comparison of the choroidal thickness of the guinea pigs after 6-week myopic induction, illustration of the measurement of ChT, and OCT fundus analysis template of guinea pigs were conducted. Structural images captured by OCT showed the structure of the defined region; the yellow lines represent the inner concentric circle (600 μm, green arrow) and outer concentric circle (1,050 μm, green arrow). **(C)** The optic disc was defined as the circle center, and the region of interest in each quadrant was located between the border of the choroidal layer and the red and blue lines. **(D)** After 6-week myopia induction, choroidal–ocular axis correlation in the NC and LIM groups, *n* = 8 **(E)**.

We measured the refractive error (Figure 1A) and axial length (Figure 1B) of both eyes of all guinea pigs before myopia induction and found no significant differences between the two groups (*p* > all 0.05). However, after 2, 4, and 6 weeks of myopia induction, we noticed that the difference in refractive error and axial length between the right and left eyes of guinea pigs in the LIM group was significantly greater (all *p* < 0.001).

#### 3.1.2 Choroidal thickness

To explore the effect of myopia induction on choroidal thickness (ChT), we further measured choroidal thickness in guinea pigs after 6 weeks of myopia induction (Figure 1C). As shown in Figures 1D,E, the results indicated that there was no significant difference in choroidal thickness between the two groups of guinea pigs before and after myopic induction (NC, 67.977 ± 3.032 μm; LIM, 67.482 ± 2.037 μm). However, 6 weeks after myopia induction we found that ChT was significantly lower in the LIM group (85.226 ± 2.13 μm) compared with the ChT value in the NC group (102.682 ± 4.87 μm).

### 3.2 Differential alterations in thiamine transporter carriers and thiamine content in myopia induction

#### 3.2.1 Q-PCR analysis

**Figure 2:**
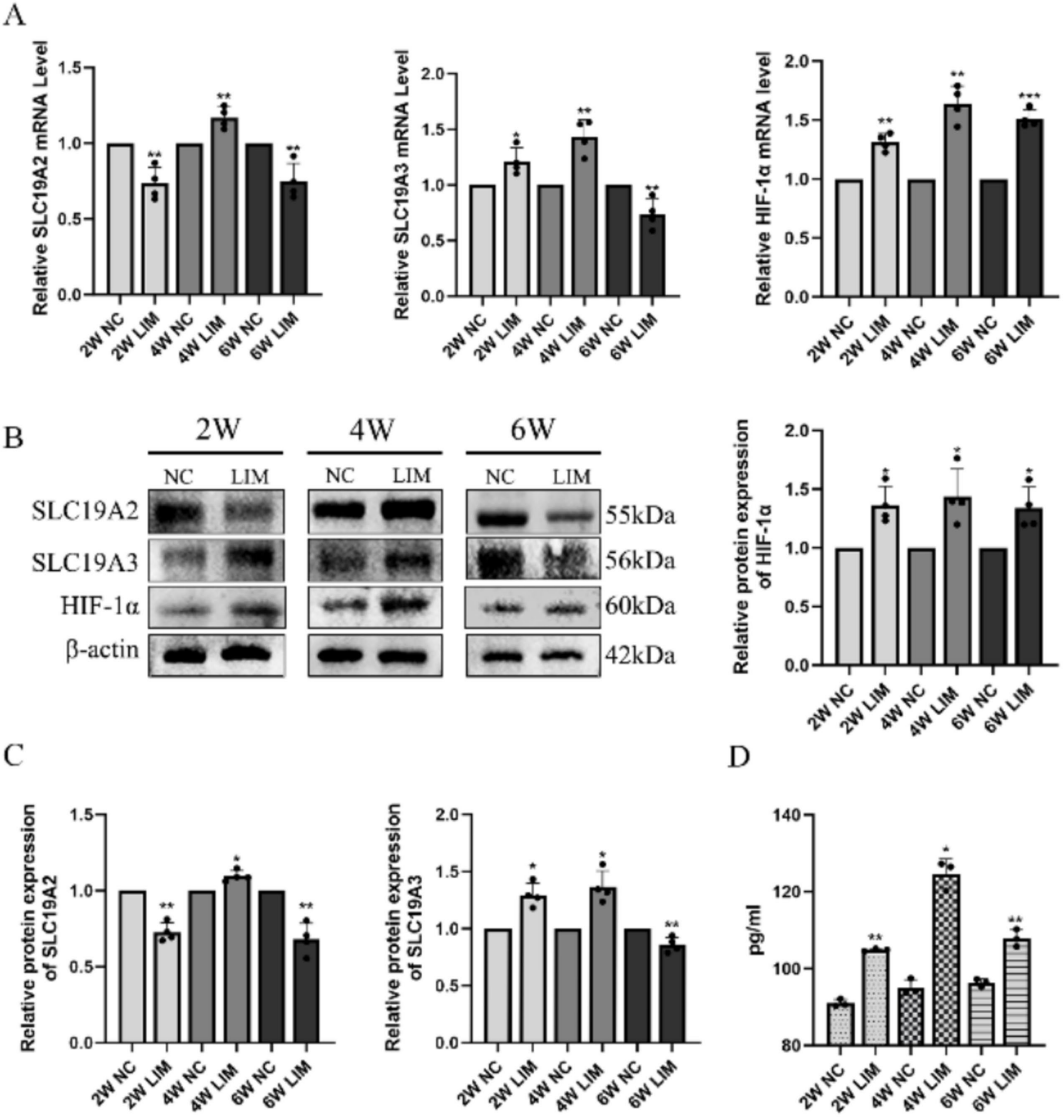
qPCR analysis of SLC19A2, SLC19A3, and HIF-1α levels in choroidal tissues after different treatments for 2, 4, and 6 weeks **(A)**. Western blot analysis of SLC19A2, SLC19A3, and HIF-1α levels in choroidal tissues in LIM guinea pigs for 2, 4, and 6 weeks **(B)**. Histogram analysis of SLC19A2, SLC19A3, and HIF-1α **(C)**. ELISA detection of thiamine in the choroid of each group **(D)**.*p < 0.05, **p < 0.01 vs. the NC group, *n* = 6.

After 2 weeks of modeling, compared with the NC group, the mRNA expression of SLC19A2 and SLC19A3 in the choroid of guinea pigs in the LIM group was significantly lower (*p* < 0.01) and higher (*p* < 0.01) than that in the NC group. After 4 weeks of modeling, the mRNA expression of SLC19A2 and SLC19A3 in the optic choroid of guinea pigs in the LIM group was significantly higher (*p* < 0.01) compared with that of the NC group, and after 6 weeks of modeling, the mRNA expression of SLC19A2 and SLC19A3 in the optic choroid of guinea pigs in the LIM group was significantly lower (*p* < 0.05) compared with that of the NC group. At the three time points, the choroidal HIF-1α mRNA expression of guinea pigs in the LIM group was significantly higher (*p* < 0.01) than that in the NC group (Figure 2A).

#### 3.2.2 Western blot analysis

After 2 weeks of modeling, the relative expression of SLC19A2, SLC19A3 and HIF-1α proteins in the choroid of guinea pigs in the LIM group was significantly lower (*p* < 0.01) and higher (*p* < 0.01) than that in the NC group. After 4 weeks of modeling, the relative expression of SLC19A2 and SLC19A3 proteins in the optic choroid of guinea pigs in the LIM group was significantly higher compared with that in the NC group (*p* < 0.01), and after 6 weeks of modeling, the relative expression of SLC19A2 and SLC19A3 proteins in the optic choroid of guinea pigs in the LIM group was significantly lower compared with that in the NC group (*p* < 0.01); after 2, 4, and 6 weeks of modeling, the relative expression of HIF-1α was all highly expressed (*p* < 0.05)(Figures 2B,C).

#### 3.2.3 Changes in thiamine levels

To explore the relationship between SLC19A2, SLC19A3, and thiamine content in the choroidal tissue of myopic guinea pigs, we examined the changes in thiamine levels at 2, 4, and 6 weeks of myopic induction. The results showed that thiamine levels in the choroidal tissue of guinea pigs in the LIM group were elevated compared with those in the NC group after myopia induction (both *p* < 0.05), with the highest-level content at 4 weeks and a decrease in levels at 6 weeks relative to 4 weeks (Figure 2D).

#### 3.2.4 Immunohistochemistry and immunofluorescence

**Figure 3:**
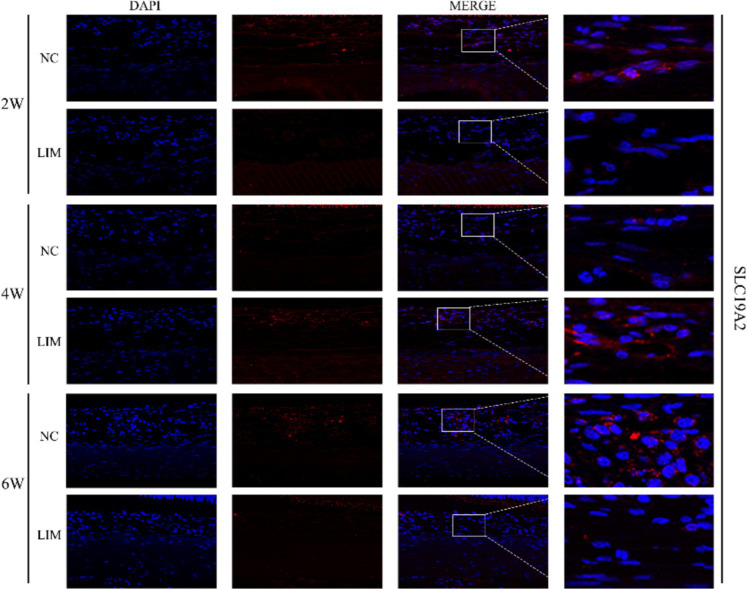
Expression of SLC19A2 in the choroid of the NC and LIM groups detected by immunofluorescence staining after 2, 4, and 6 weeks of myopia induction. Magnification: 100×. Results detected by immunohistochemistry after 2, 4, and 6 weeks of myopia induction, *n* = 3.

**Figure 4:**
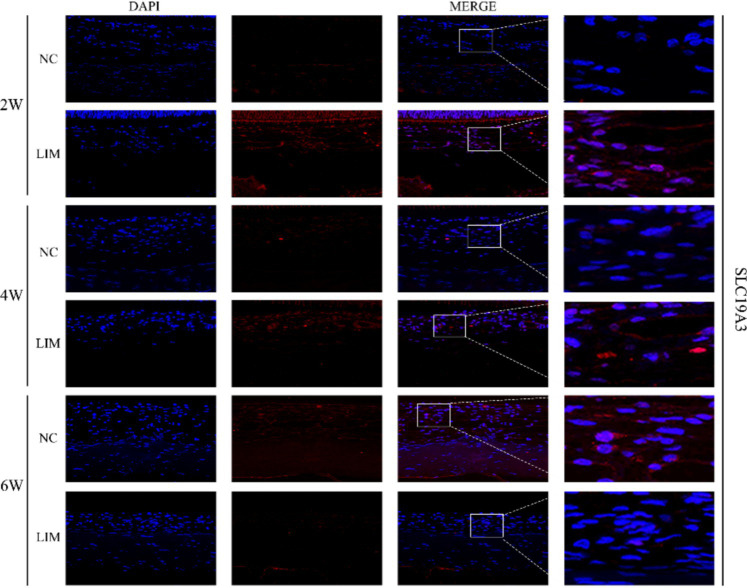
Expression of SLC19A3 in the choroid of the NC and LIM groups detected by immunofluorescence staining after 2, 4, and 6 weeks of myopia induction. Magnification: 100×. Results detected by immunohistochemistry after 2, 4, and 6 weeks of myopia induction, *n* = 3.

**Figure 5:**
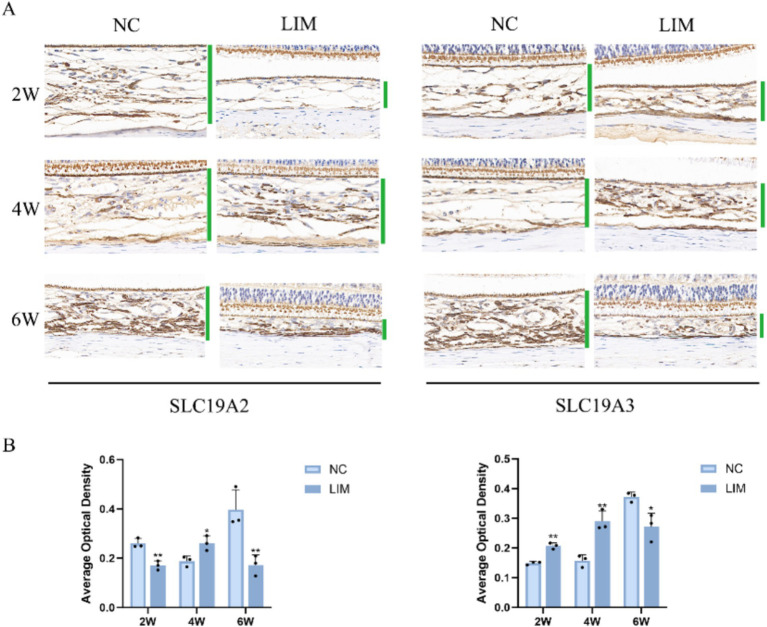
Results detected by immunohistochemistry after 2, 4, and 6 weeks of myopia induction. **(A)** Magnification: 50×. Average optical density analysis of SLC19A2 and SLC19A3 **(B)**. *p < 0.05, **p < 0.01 vs. the NC group, *n* = 3.

To determine the expression levels of SLC19A2 (Figure 3) and SLC19A3 (Figure 4) in the choroidal tissues of myopic guinea pigs at different myopia induction times, we performed immunofluorescence staining of the choroidal tissues of myopic guinea pigs. As shown, DAPI-stained nuclei showed blue color under UV excitation and were positively expressed in red color labeled with the corresponding fluorescein. The fluorescence intensity of SLC19A3 was stronger in the choroidal tissues of guinea pigs in the 2- and 4-week LIM group than that of the NC group, whereas the fluorescence intensity of SLC19A2 was stronger in the choroidal tissues of guinea pigs in the 4-week LIM group than that of the NC group only. Immunohistochemistry results were consistent with immunofluorescence results after mean optical density analysis (Figure 5).

### 3.3 Myopic choroidal hypoxia causes changes in thiamine transporter carriers due to HIF-1α interaction with SLC19A3

#### 3.3.1 Histopathologic staining

**Figure 6:**
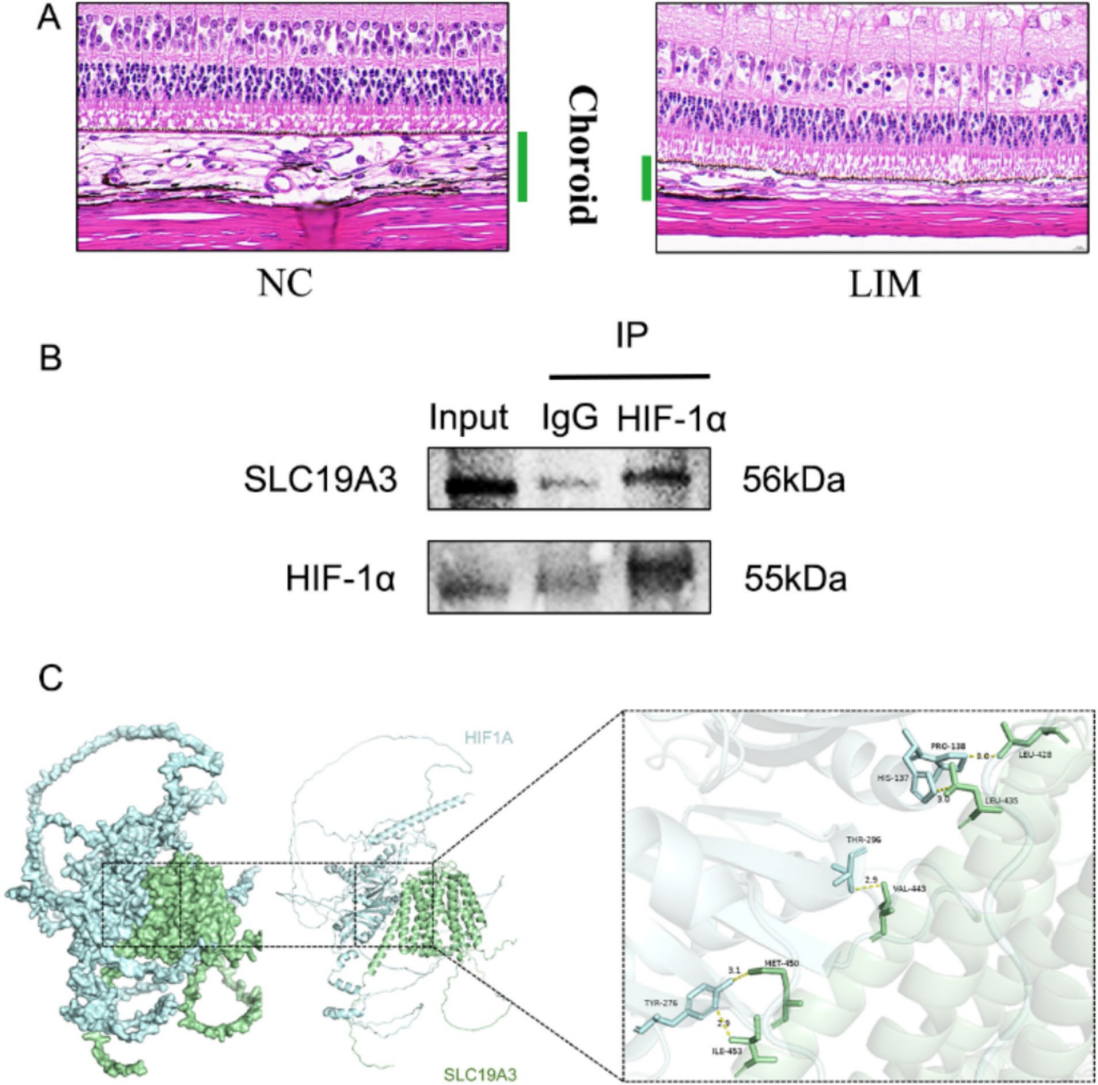
Histopathological assessment of the eye tissues after 6-week myopic induction **(A)**.Co-IP for detection of direct interaction of HIF-1α with SLC19A3 **(B)**. Molecular docking to observe the binding of HIF-1α to SLC19A3. **(C)** Docking score: −318.58, Confidence Score:0.9668, Ligand RMSD:52.47.

H&E staining of ocular tissue sections showed the histopathological characteristics of the choroid of 6-week-old guinea pigs. The results showed that the structural arrangement of the choroid was densely organized in the NC group, and the capillary structure was more tightly structured than in the LIM group, while the structural arrangement within the interstitium of the choroid was more lax in the LIM group, and the fracture lysis was more pronounced and sparse (Figure 6A).

#### 3.3.2 Protein immunoprecipitation and molecular docking

We performed protein immunoprecipitation of HIF-1α and SLC19A3 to investigate whether they interact. The results of the experiment showed that both HIF-1α protein and SLC19A3 protein were expressed in the Input group, and there was no significant protein expression in the negative control group of IgG, but the interaction between the two proteins was proved in the IP group (Figure 6B). After proving the interaction between the two proteins, we observed the binding between the two proteins by molecular docking, hydrogen bonding is shown in yellow, and HIF-1α and SLC19A3 are shown in blue and green, respectively (Figure 6C).

## 4 Discussion

Myopia has become one of the most serious public health problems in the world with the increase in the amount of time people spend working in close proximity and the prolonged use of electronic devices (18), and although the exact mechanisms underlying the development of myopia are unknown, there is growing evidence that the choroid may contribute to the development of myopia.

The choroid is the posterior portion of the uvea, located between the retinal pigment epithelium and the sclera, and extends from the serosa forward to the optic nerve extending backward, and consists of blood vessels, melanocytes, fibroblasts, resident immunoreactive cells, and supporting collagenous and elastic connective tissue (18). The main functions of the choroid are to provide oxygen and nutrients to the outer retina, to reposition the retina through changes in thickness, and to release growth factors involved in the regulation of blood vessel formation and scleral remodeling as well as in the regulation of eye growth (19). It has been shown that decreased choroidal blood perfusion (ChBP) leads to scleral hypoxia and fibroblast transdifferentiation thereby contributing to myopia (20), and changes in choroidal thickness also show a positive correlation with myopia (21, 22).

Research has demonstrated that the interconversion of pentose and glucuronic acid in the atrial fluid, as well as the citric acid cycle (TCA cycle), is significantly altered in patients with pathological myopia caused by choroidal neovascularization (23). Additionally, pyruvate metabolism in the choroidal tissues of myopic rabbits has been found to be abnormal (24). Thiamine, also known as vitamin B1, is a water-soluble vitamin that plays a crucial role in gluconeogenesis, oxidative metabolism, ATP generation and reduction, as well as in managing cellular oxidative stress. It is involved in the oxidative decarboxylation of pyruvate and contributes to the pentose phosphate pathway within the TCA cycle. Therefore, thiamine may be considered to have an important role in relation to myopia. The two transporters THTR1 and THTR2, which are mainly responsible for thiamin transport across the plasma membrane, are encoded by the SLC19A2 and SLC19A3 genes, respectively (25), with THTR-2 having a significantly higher affinity for thiamin (26), which was also verified in our study.

In the present study, we found that with the increase of myopia induction time, thiamine content in guinea pig choroidal tissues was elevated at the highest level at 4 weeks, and thiamine level decreased but was still higher than that of control group at 6 weeks of modeling; according to the mRNA and protein expression levels, SLC19A3 transporter protein was firstly highly expressed at 2 weeks, and then both SLC19A2 and SLC19A3 were highly expressed at 4 weeks, and then lowly expressed at 6 weeks, suggesting that thiamine was co-regulated by SLC19A2 and SLC19A3 is even more obvious. This may be related to the fact that the minor structural differences of SLC19A3 compared to SLC19A2 result in a greater affinity for thiamine, leading to differences between the two transporter proteins (27).

It has been shown that the up-regulation of SLC19A3 is associated with the direct binding of HIF-1α to it and activation of its expression under hypoxic stress conditions (28, 29), and the up-regulation of HIF-1α expression has also been observed in other studies on myopic choroidal tissues (30). In our study, the expression of HIF-1α was high at 2, 4, and 6 weeks of myopia induction. Moreover, through CO-IP and molecular docking, it was demonstrated that HIF-1α could directly bind and activate SLC19A3 expression, which indicated that the elevation of thiamine in myopic choroidal tissues and the different trends of SLC19A3 and SLC19A2 were not only affected by the structure of its transporter protein but also inseparably related to the activation of SLC19A3 expression by HIF-1α. So we believe that this is an adaptive response process of tissues to fight against changes in the external environment.

New blood vessels in the choroid can grow into the normally avascular outer retinal layer and subretinal space, and oxidative stress in the retinal pigment epithelium and photoreceptors leads to higher levels of HIF-1α. In pathological myopia, lengthening of the ocular axis leads to a reduction in retinal vascularization and to stenosis and other vascular alterations that produce oxidative stress (31), whereas retinal blood supply oxygenation is generated by choroidal blood flow, and choroidal thinning is also observed in myopic tissues, and these results also reflect circulatory disturbances in the development of myopia. This relationship has also been confirmed in animal models of myopia (32). During myopia, as the eye axis grows, the thinning of choroidal thickness decreases blood perfusion resulting in a hypoxic environment (33), which produces oxidative stress in myopia induction (34), increased generation of oxygen free radicals in tissues, and the subsequent direct binding of HIF-1α expression to activate the expression of SLC19A3, which in turn increases thiamine transport, which serves as an important cofactor for the transketolase in the pentose phosphate pathway (PPP) Thiamin acts as an important cofactor in the transketolase reaction of the pentose phosphate pathway (PPP) to produce NADPH and glutathione for the biosynthetic pathway to protect the redox balance of the cellular tissues (35), to resist apoptosis due to excessive oxidative stress and glutathione depletion, and to play the role of antioxidant, which reflects the adaptive regulation of tissues to changes in the external environment. However, myopia induction is a persistent pathological process, even though increased thiamine transport can antagonize oxidative stress, it ultimately fails to alleviate the process of pathologic myopia, myopia continues to progress, and the role of thiamine in the process of myopia on the specific molecular mechanisms needs to be further investigated.

The results showed that thiamine changes in the choroidal tissues of myopic guinea pigs were associated with HIF-1α-mediated expression of SLC19A3, suggesting an adaptive regulatory process of thiamine in the myopic choroid, and are expected to provide new insights for the study of molecular mechanisms of myopia.

## Data Availability

The datasets presented in this study can be found in online repositories. The names of the repository/repositories and accession number(s) can be found in the article/Supplementary material.

## Footnotes

^1^ http://hdock.phys.hust.edu.cn/
