# Supplementary figures and images for: Elevated thiamine level is associated with activating interaction between HIF-1α and SLC19A3 in experimental myopic guinea pigs
Source: Front Med (Lausanne). 2025 Apr 25;12:1503527. doi: 10.3389/fmed.2025.1503527 (PMC12061867; doi:10.3389/fmed.2025.1503527)

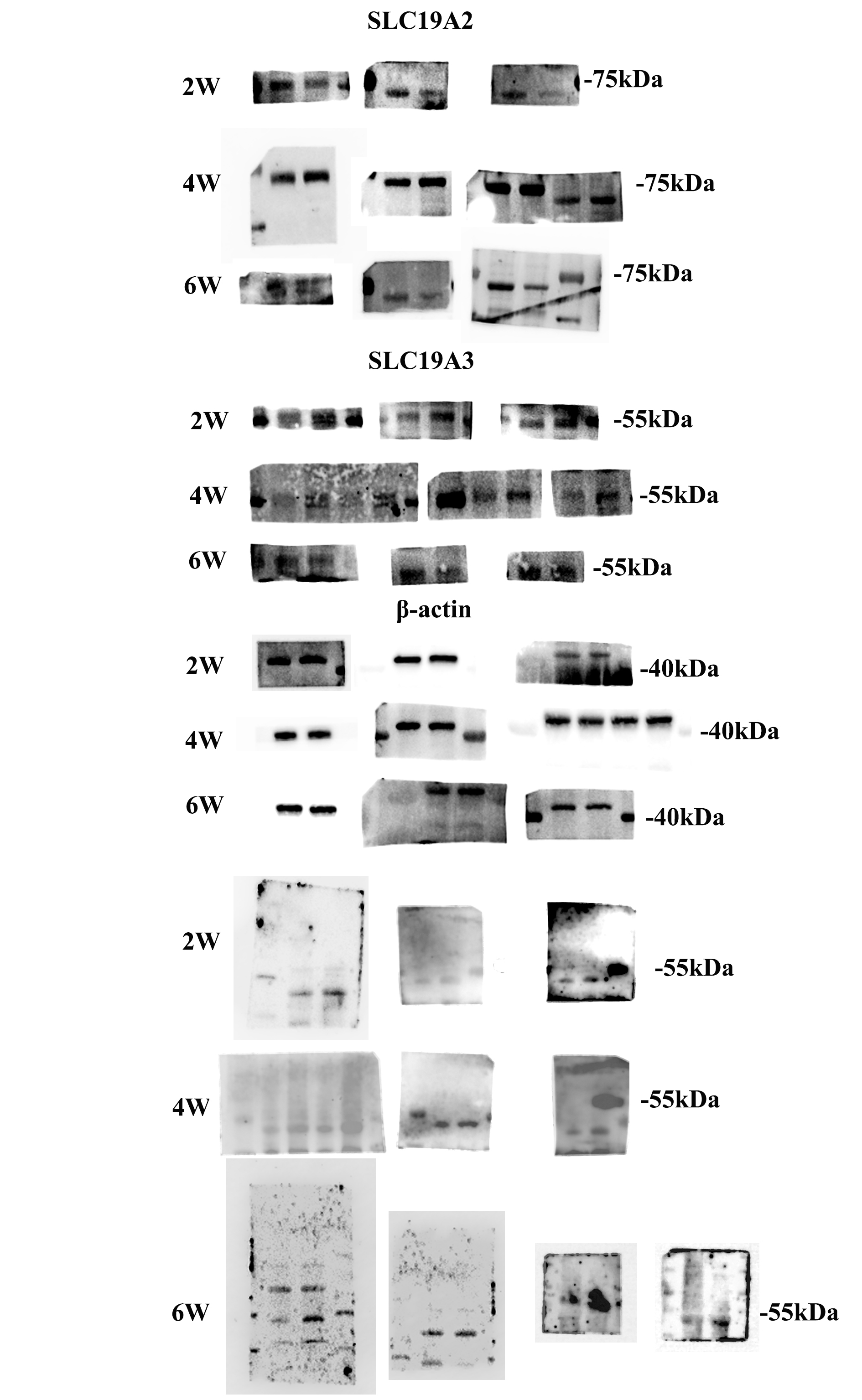

Supplement: Supplementary file 1 [file Image_1.jpeg]
